# 3D-Printed Brace Utilization for an Isolated Fibular Head Fracture: A Case Report

**DOI:** 10.7759/cureus.79220

**Published:** 2025-02-18

**Authors:** Ji Lee, Vincent Lee, Rana Haggag, Kenny Brock, Michael Goodlett

**Affiliations:** 1 Medicine, Edward Via College of Osteopathic Medicine, Auburn, USA; 2 Research and Immunology, Edward Via College of Osteopathic Medicine, Auburn, USA; 3 Sports Medicine, Auburn University, Auburn, USA

**Keywords:** 3d-printed brace, case report, fibular head, fracture, mri, x-ray

## Abstract

Isolated fibular head fractures are rare and often result from direct trauma to the lateral knee or varus stress. These fractures, accounting for a small percentage of fibular fractures, can be challenging to diagnose on initial imaging. We present a case of a 65-year-old man who sustained a blow to the right fibular head from a cow's kick. Initial X-rays were inconclusive, but an MRI revealed an impacted fibular head fracture. The patient was managed conservatively with a 3D-printed brace, a walking boot, and crutches for mobility support. The brace, made of carbon fiber nylon with a thickness of 4 mm, was secured using an elastic bandage and rubber adhesive. It provided lateral stability and pressure to the tibial head, improving the patient's perceived stability while walking. The patient recovered fully without complications. Unlike fractures of the distal fibula, which often require surgical intervention, fibular head fractures rarely need surgery and can be managed effectively with conservative measures. This case highlights the value of advanced imaging and customized 3D-printed bracing in managing isolated fibular head fractures.

## Introduction

Fibula fractures typically result from traumatic events, often involving the twisting of the leg or foot or direct impact on the lateral leg. These fractures rarely occur in isolation and are frequently associated with injuries to adjacent structures, including the proximal tibia, knee ligaments, meniscus, and neurovascular components such as the common peroneal nerve. In cases of ankle fractures or sprains, the energy from the injury can sometimes travel up the leg, resulting in a fibula fracture. Among ankle fractures, distal fibula fractures are the most common type [1]. Isolated fibula fractures, without any associated injuries to the ankle or knee bones or ligaments, are typically found in the diaphysis or distal third of the bone. However, isolated fractures of the proximal fibular head are extremely rare, accounting for approximately 2.05% of all fibular fractures [2]. These injuries are generally caused by direct impact to the fibular head or varus stress on the knee. The severity of the fracture can vary depending on whether it is displaced or non-displaced. Following a high-impact injury with rapid and significant swelling, prompt evaluation for compartment syndrome is essential to prevent serious complications. Untreated compartment syndrome can lead to irreversible muscle and nerve damage, kidney failure, or systemic infections [3]. The lower limb is divided into four compartments: anterior, lateral, superficial posterior, and deep posterior. The fibular head lies within the lateral compartment, alongside the common peroneal nerve, which branches into the superficial and deep peroneal nerves. Trauma to the lateral knee necessitates neurovascular evaluation due to the superficial location of the peroneal nerve. Damage to the common peroneal nerve may present as numbness and tingling on the lateral leg, the dorsum of the foot, and the webbed space between the first and second toes. The hallmark signs of common peroneal nerve damage include weakened dorsiflexion, resulting in foot drop, and sensory loss over the first dorsal web space, warranting the evaluation of the fibular head given the nerve's proximity to this area [4]. Here, we present a case of a traumatic, isolated fibular head fracture managed non-operatively with a 3D-printed brace applied over the fracture site, in combination with a standard walking boot.

## Case presentation

**Figure 1:**
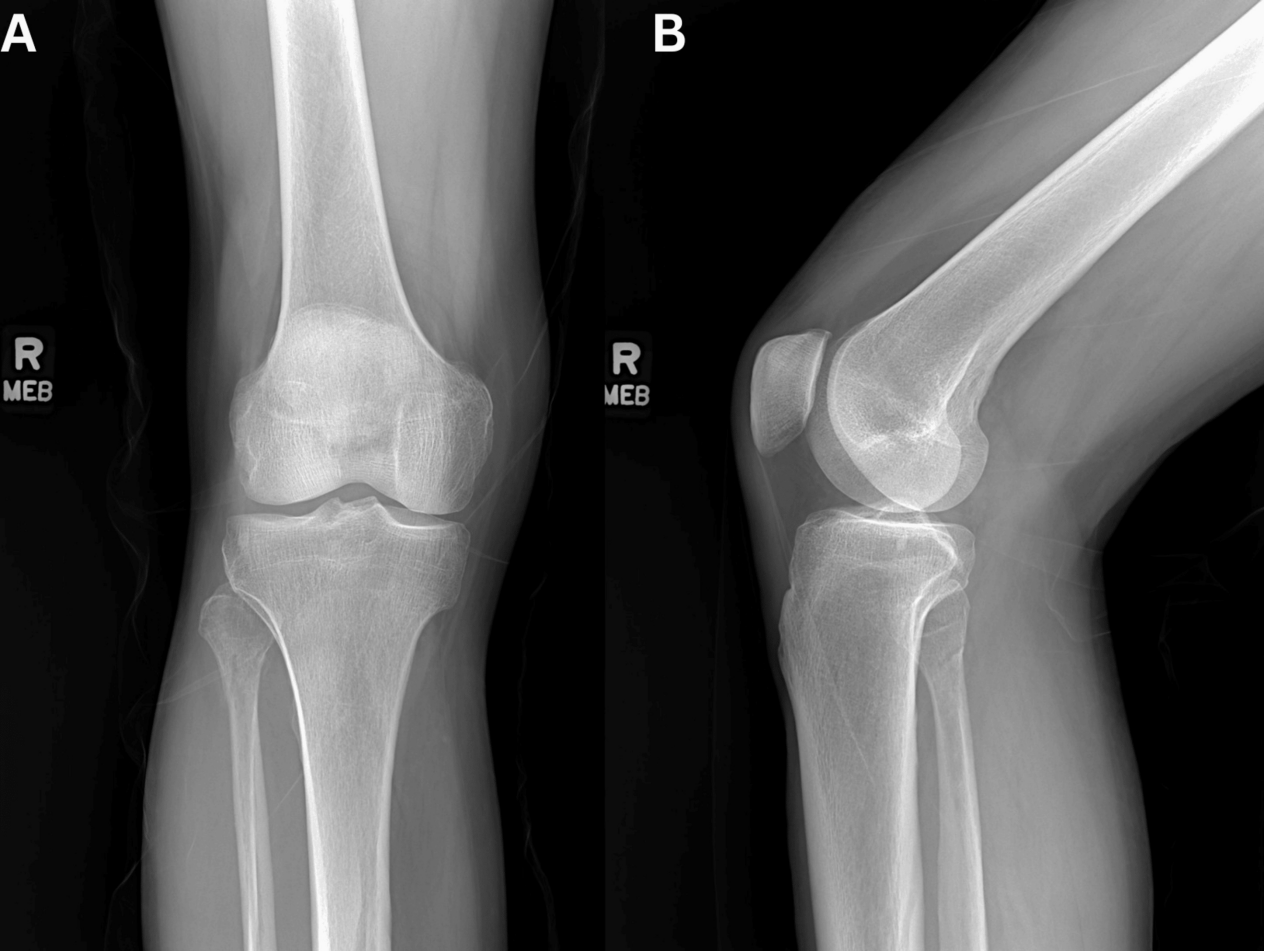
Initial X-rays of the right knee. (A) Anteroposterior and (B) lateral X-ray views of the right knee, taken approximately 24 hours after the injury, showing no fractures or abnormalities.

A 65-year-old man presented to urgent care following an injury to the right proximal fibula caused by a cow's kick to the lateral knee. The patient reported immediate and significant swelling in the area. Initial X-rays showed no abnormalities (Figure 1).

Five days later, the patient sought care from another physician due to ongoing swelling and pain despite rest and ice application. On physical examination, the palpation of the posterior right proximal fibula caused a tingling sensation, but there were no signs of foot drop or other nerve damage. Although no significant findings were present, the patient continued to experience considerable pain, prompting the decision to pursue more advanced imaging. The subsequent MRI revealed an impacted fracture of the right proximal fibular head and also showed joint effusion.

**Figure 2:**
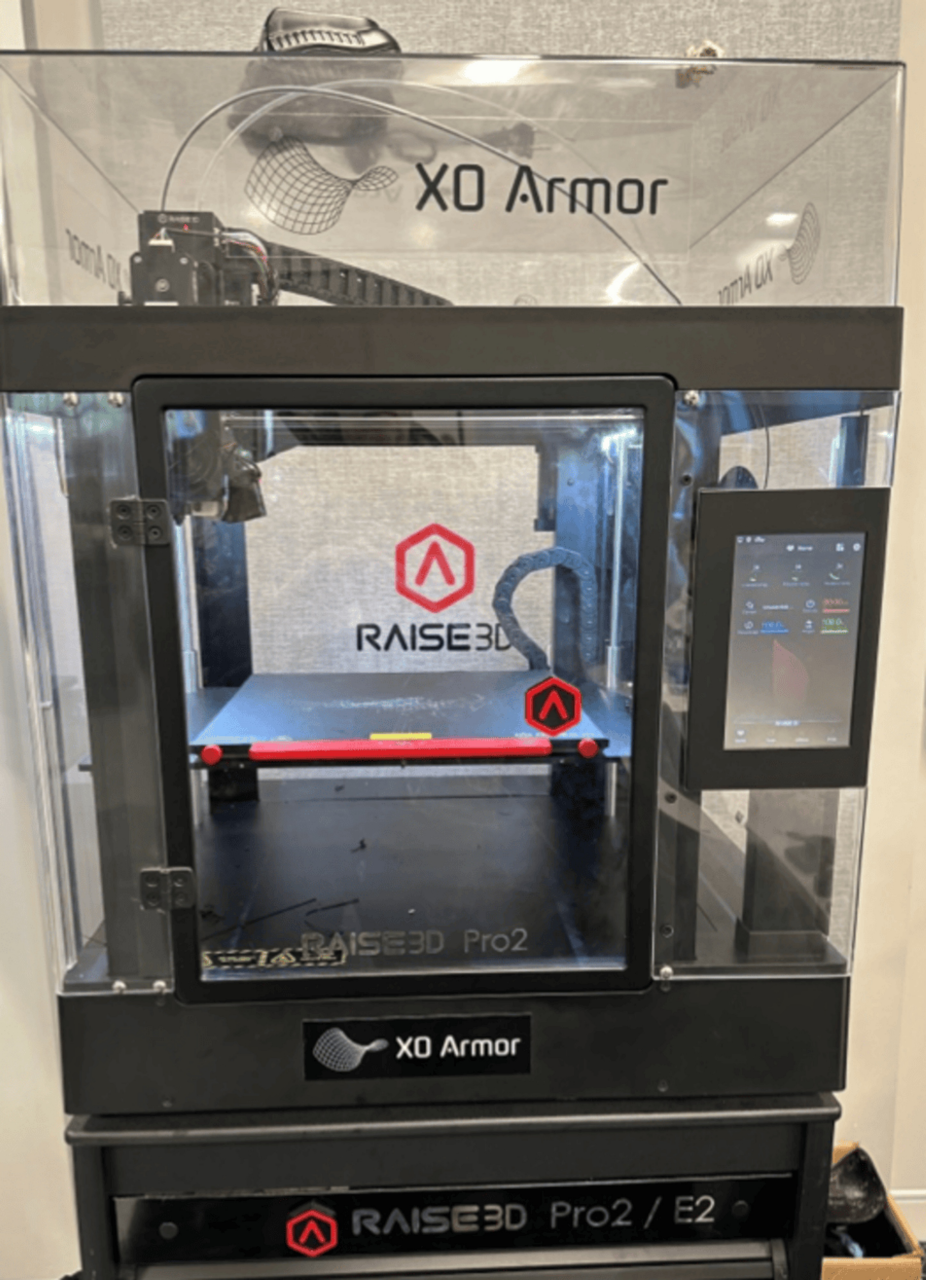
XO Armor 3D printing machine. XO Armor is a high-tech, durable medical equipment manufacturing company.

**Figure 3:**
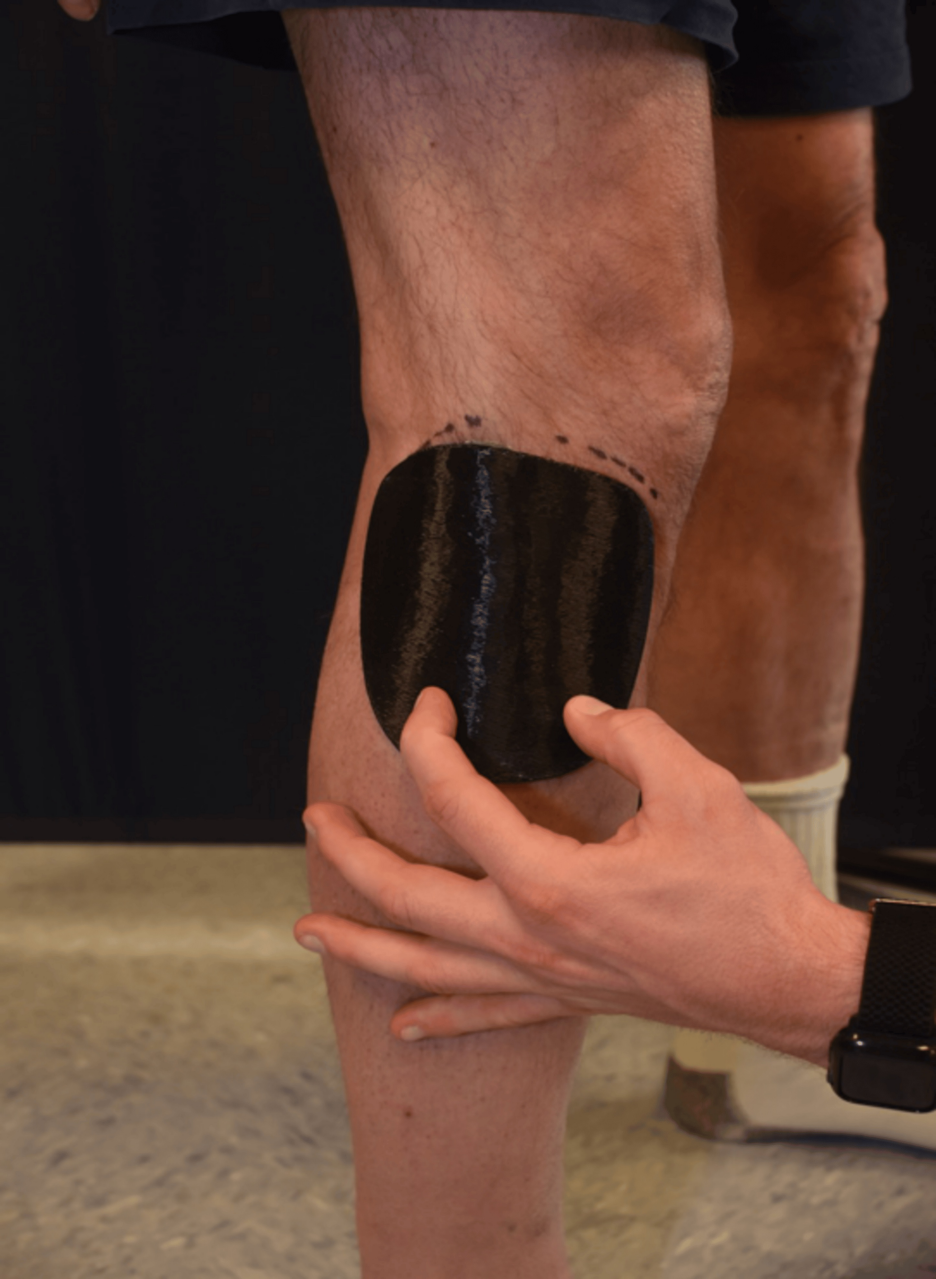
3D-printed brace for fibular head fracture. The brace is applied to the fibular head for support and stabilization. The ace wrap, used to secure the brace, is not shown in this image to provide clarity of the brace itself.

**Figure 4:**
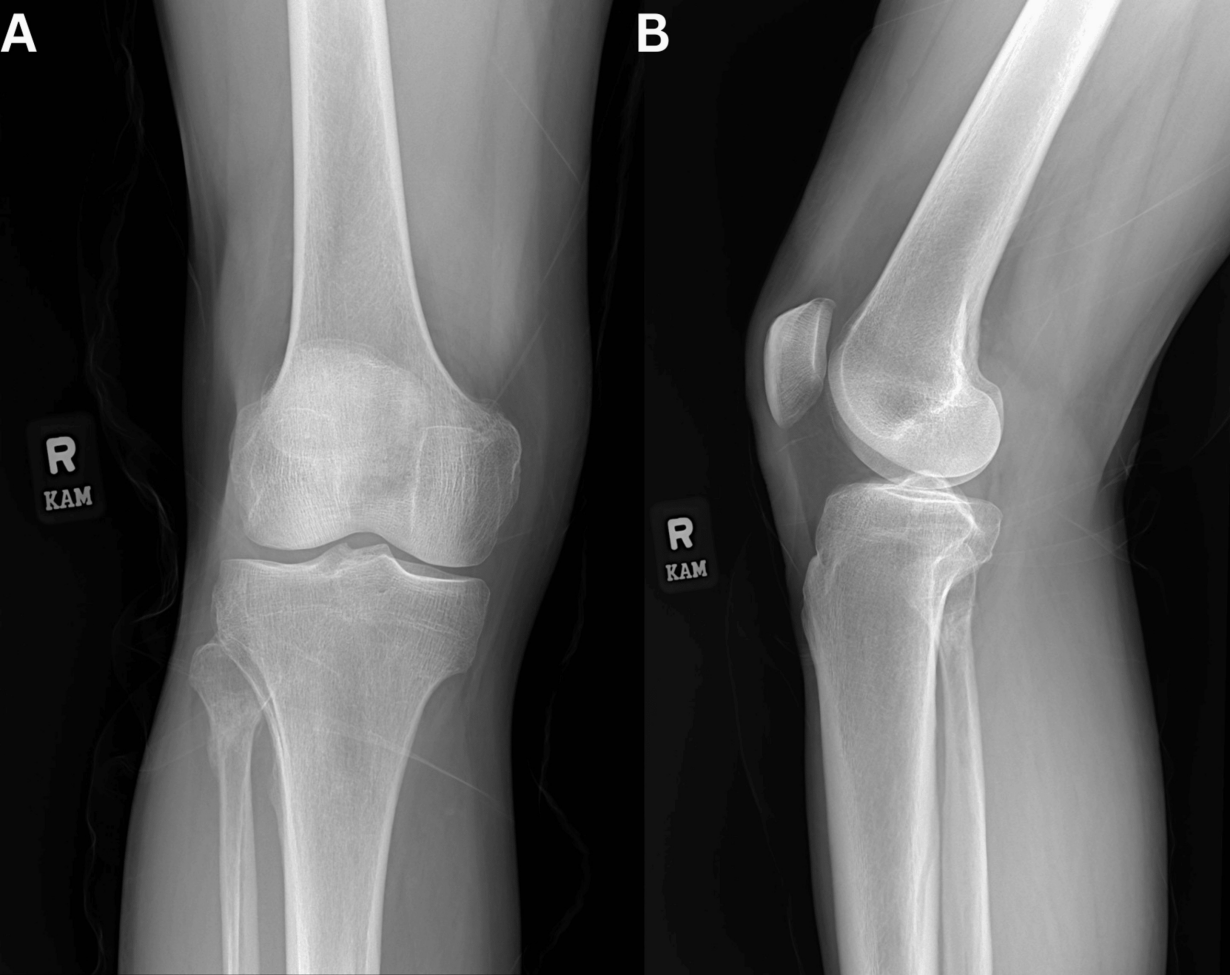
Final follow-up X-ray of the right knee. (A) Anteroposterior and (B) lateral X-ray views of the right knee, taken 10 weeks after the injury, showing bridging callus formation across the proximal fibular fracture. The healing fracture is in normal alignment.

Since the fracture was neither significantly displaced nor comminuted, non-operative management was deemed appropriate. A 3D-printed brace, created using the XO Armor 3D printing machine (Auburn, AL) (Figure 2), was recommended for recovery, alongside a standard walking boot and crutches. XO Armor is a medical equipment manufacturing company whose 3D printers are often used to create custom-fit protective gear for athletes and patients. The brace was considered a superior alternative to traditional full-leg casting or splinting, which were unnecessary for this injury. It was mainly designed to provide lateral stabilization of the fibular head, protect the fractured area from further trauma, reduce swelling and pain, and offer a compact, targeted solution (Figure 3). The brace was well-tolerated and used in conjunction with a walking boot, offering greater mobility and comfort during recovery compared to using a full cast or immobilizing the entire leg. Follow-up X-rays at 10 weeks post-injury demonstrated proper bone healing and alignment (Figure 4). The patient achieved full recovery without any long-term complications, resuming daily activities and expressing satisfaction with the functional outcome.

## Discussion

The utilization of a 3D-printed brace for an isolated fibular head fracture offers a novel approach to addressing a key challenge in conservative management: providing localized support and protection without restricting knee joint mobility. While traditional braces typically aim to immobilize one or more joints, the purpose of the brace in this case was to offer targeted lateral stabilization and pressure distribution to the fibular head. This stabilization was essential in supporting the fracture site and preventing excessive movement of the fibula, which could potentially aggravate the injury or delay recovery. By stabilizing the fibula along the outside of the leg, the brace ensured protection without interfering with the natural range of motion in the knee, minimizing discomfort and enhancing the patient's sense of stability during walking.

Once the brace was applied, the patient reported immediate improvements in walking stability and a notable reduction in pain. This response indicated the brace's effectiveness in improving mobility and managing pain, and the patient continued to use it throughout the remainder of the recovery period. Constructed with carbon fiber nylon, the brace was designed to offer flexibility and durability while remaining lightweight. The patient used a 4 mm-thick version of the brace, though an alternative 8 mm-thick version was also available. To secure the brace, an elastic bandage (vet wrap) and ace wrap were used, providing a snug yet adjustable fit. The choice of carbon fiber nylon was intentional, as it balances rigidity with adaptability, allowing the brace to contour to the patient's leg without causing excessive pressure or discomfort.

A secondary consideration in the brace's design was its ability to shield the fibular head from incidental trauma. The primary objective of the brace, however, was to provide lateral stabilization to the leg area below the knee joint, specifically around the fibular head. While the brace is not intended to immobilize the knee joint, it serves as a protective barrier against minor impacts that could otherwise delay recovery. The rubber adhesive layer, incorporated on the inside of the brace, enhances its ability to absorb and distribute force, reducing localized stress on the fracture site. This inner layer provides a cushioning effect, helping to minimize the impact of external forces on the fibula while maintaining the brace's flexibility and stability. This feature proved beneficial, as the patient reported an increased sense of stability while walking, further supporting the clinical utility of the device in conservative management. Unlike traditional splints or casts, which could compromise knee mobility and increase the risk of complications such as joint stiffness, muscle atrophy, or compromised circulation, this brace provided an alternative method of external support without restricting necessary motion [5].

Immobilizing the knee completely would have been excessive for a non-displaced fibular head fracture, given that the fibular head itself does not bear weight during ambulation [6]. Instead, the brace's lateral stability mechanism contributed to a more controlled and comfortable recovery process. Diagnosing fibular head fractures on initial X-rays can be challenging, as subtle or non-displaced fracture lines may not be immediately evident, especially in the absence of associated injuries. X-rays are better suited for detecting fractures with clear cortical disruption, whereas advanced imaging modalities such as MRI can provide a more detailed evaluation, identifying subtle fracture lines and associated soft tissue injuries [7]. Current literature on brace utilization for fibular head fractures is nonexistent, as standard management typically involves rest, pain control, and a gradual return to activity without the use of external braces. Traditional treatments for fibular head fractures generally prioritize minimizing interference with knee joint mobility, given the fracture's location just below the knee joint. The use of splints or casts was not considered in this case, as immobilizing the knee joint could impede motion and potentially lead to complications such as joint stiffness, muscle atrophy, or neurovascular compromise, particularly involving the common peroneal nerve [8]. This gap in the literature underscores the novelty of using a 3D-printed device to provide localized support and lateral stabilization. This case thus presents a promising alternative to traditional methods and suggests the potential of 3D-printed braces to address specific biomechanical concerns in fibular head fractures. Future studies are needed to explore the role of orthotic devices in managing these fractures and determine optimal brace designs and materials to improve patient outcomes.

## Conclusions

In conclusion, while the brace did not directly facilitate fracture healing, it provided targeted support and protection, contributing to a more stable and comfortable recovery process. The case underscores the potential for customized 3D-printed braces to supplement traditional conservative management strategies for fibular head fractures, warranting further investigation into their broader clinical applications.

## References

[1] Aiyer AA, Zachwieja EC, Lawrie CM, Kaplan JR (2019). Management of isolated lateral malleolus fractures. J Am Acad Orthop Surg.

[2] Havránek P (1996). Proximal fibular physeal injury. J Pediatr Orthop B.

[3] Tillinghast CM, Gary JL (2019). Compartment syndrome of the lower extremity. Compartment Syndrome.

[4] Lezak B, Massel DH, Varacallo MA (2024). Peroneal Nerve Injury. StatPearls - NCBI Bookshelf. Published Online First: 14 November.

[5] Christensen B, Dyrberg E, Aagaard P, Kjaer M, Langberg H (2008). Short-term immobilization and recovery affect skeletal muscle but not collagen tissue turnover in humans. J Appl Physiol (1985).

[6] Vance DD, Vosseller JT (2017). Double plating of distal fibula fractures. Foot Ankle Spec.

[7] Jarraya M, Hayashi D, Roemer FW (2013). Radiographically occult and subtle fractures: a pictorial review. Radiol Res Pract.

[8] Baima J, Krivickas L (2008). Evaluation and treatment of peroneal neuropathy. Curr Rev Musculoskelet Med.

